# Radiomics signature on CECT as a predictive factor for invasiveness of lung adenocarcinoma manifesting as subcentimeter ground glass nodules

**DOI:** 10.1038/s41598-021-83167-3

**Published:** 2021-02-11

**Authors:** Wufei Chen, Ming Li, Dingbiao Mao, Xiaojun Ge, Jiaofeng Wang, Mingyu Tan, Weiling Ma, Xuemei Huang, Jinjuan Lu, Cheng Li, Yanqing Hua, Hao Wu

**Affiliations:** 1 Department of Radiology, Huadong Hospital Affiliated To Fudan University, 221 West Yan’an Road, Shanghai, 200040 China; 2 Department of Science and Education, Huadong Hospital Affiliated To Fudan University, 168 West Yan’an Road, Shanghai, 200040 China; 3 Diagnosis and Treatment Center of Small Lung, Nodules of Huadong Hospital, Shanghai, China

**Keywords:** Non-small-cell lung cancer, Image processing

## Abstract

Controversy and challenges remain regarding the cognition of lung adenocarcinomas presented as subcentimeter ground glass nodules (GGNs). Postoperative lymphatic involvement or intrapulmonary metastasis is found in approximately 15% to 20% of these cases. This study aimed to develop and validate a radiomics signature to identify the invasiveness of lung adenocarcinoma appearing as subcentimeter ground glass nodules. We retrospectively enrolled 318 subcentimeter GGNs with histopathology-confirmed adenocarcinomas in situ (AIS), minimally invasive adenocarcinomas (MIA) and invasive adenocarcinomas (IAC). The radiomics features were extracted from manual segmentation based on contrast-enhanced CT (CECT) and non-contrast enhanced CT (NCECT) images after imaging preprocessing. The Lasso algorithm was applied to construct radiomics signatures. The predictive performance of radiomics models was evaluated by receiver operating characteristic (ROC) analysis. A radiographic-radiomics combined nomogram was developed to evaluate its clinical utility. The radiomics signature on CECT (AUC: 0.896 [95% CI 0.815–0.977]) performed better than the radiomics signature on NCECT data (AUC: 0.851[95% CI 0.712–0.989]) in the validation set. An individualized prediction nomogram was developed using radiomics model on CECT and radiographic model including type, shape and vascular change. The C index of the nomogram was 0.915 in the training set and 0.881 in the validation set, demonstrating good discrimination. Decision curve analysis (DCA) revealed that the proposed model was clinically useful. The radiomics signature built on CECT could provide additional benefit to promote the preoperative prediction of invasiveness in patients with subcentimeter lung adenocarcinomas.

## Introduction

The development of computed tomography (CT) and widespread implementation of lung cancer screening programs has led to a frequently reported incidence of small-sized lung adenocarcinomas presented as ground glass nodules (GGNs)^1^. As suggested by the eighth version of the TNM classification of lung cancer, the prognosis of small lung cancer is significantly different depending on tumor size. Even c-T1 (subcentimeter) lung cancers do not always indicate early stage^2^. Postoperative lymphatic involvement or intrapulmonary metastasis is found in approximately 15% to 20% of these cases^3, 4^. However, controversy and challenges remain regarding the cognition of these lesions.

According to the classification proposed by the International Association for the Study of Lung Cancer/American Thoracic Society/European Respiratory Society in 2011^5^, cases of lung adenocarcinomas with pure lepidic growth pattern (adenocarcinomas in situ, AIS) and the invasive area less than 5 mm (minimally invasive adenocarcinomas, MIA) have a good prognosis after surgical resection. In this study, we regard these two subtypes as non-invasive group. While those with invasive foci larger than 5 mm (invasive adenocarcinomas, IAC) are associated with a risk of recurrence via lymphatic, vascular and pleural invasion, thus have a poor outcome for patients^6^. It is therefore important to meticulously diagnose invasiveness of lung adenocarcinomas for personalized clinical decision.

Radiomics analysis could assess the intratumoral biological heterogeneity using a large number of high dimensional mineable features extracted from imaging data mathematically, thereby could give an important prognostic information regarding the differentiation of benign and malignant tumors, and to assess tumor microenvironment^7–9^. It has revealed potential medical application values for evaluating the invasiveness of lung cancer in many studies^10, 11^. However, most of the previous researches extracted radiomics features from non-contrast enhanced CT (NCECT) images. The efficiency of radiomics analysis based on contrast-enhanced CT (CECT) images, which could provide additional intelligence on the intratumoral physiology of blood supply, is less commonly reported.

We hypothesize that this information could reflect by radiomics analysis, thus giving rise to a better prediction model. Hence, the aim of this study was to develop a radiomics approach based on CECT to differentiate invasive lung adenocarcinomas from non-invasive ones in patients with subcentimeter GGNs.

## Materials and methods

### Ethical approval

This retrospective study was approved by the institutional review board of Huadong Hospital. Patients’ informed consent was performed under a waiver of the authorization for that the retrospective research using anonymous data. All procedures were performed under the relevant guidelines and regulations.

### Study population

Between January 2014 and August 2018, we retrospectively evaluated 1625 patients with surgically resected c-T1a lung adenocarcinomas through pathology databases and transverse CT images in our hospital. The inclusion criteria were as follows: (1) adenocarcinomas manifested as GGNs on lung window setting (level, − 500 Hounsfield unit [HU]; width, 1500 HU); (2) thin-sections (1–1.25 mm) NCECT and CECT scans were obtained at one examination; (3) lesion ≤ 1 cm in axial CT images. The exclusion criteria included (1) histologic diagnosis other than adenocarcinoma (e.g. atypical adenomatous hyperplasia or squamous cell carcinoma); (2) noticeable motion artifacts on CT images; (3) adenocarcinoma manifested as a solid nodule. Finally, 318 subcentimeter GGNs in 318 patients fulfilled the criteria and were randomly assigned to the training set and the validation set.

### CT scan parameters

CT examinations were performed with one of the two scanners: GE Discovery CT750 HD scanner (GE Healthcare, USA) and Somatom Definition flash (Siemens Medical Solutions). All patients were asked to hold the breath at the end of inspiration. NCECT images were acquired in the supine position. The details of the scanning parameters were as follows: tube voltage 120kVp, tube current 120-200 mA, collimation 0.6 or 0.625 mm*64, rotation time 0.33 or 0.5 s/rot, SFOV 50 cm, slice thickness of reconstruction 1or 1.25 mm, slice interval of reconstruction 1or 1.25 mm, reconstruction algorithm STND and Medium sharp, matrix 512 × 512. After NCECT scanning, a dose of 80–100 ml non-ionic IV contrast material (350 mg iodine/ ml, Optiray, Mallinckrodt) was injected into the antecubital vein at a rate of 3.0–4.0 ml/s using a powerful automated injector. The CECT scanning was performed at 35 to 60 s after the injection.

### VOI segmentation and radiographic characteristics assessment

The 3D VOI was segmented manually layer-by-layer by one experienced thoracic radiologist on NCECT and CECT raw DICOM format images.The radiologist was blinded to the histopathological results of the tumors. Large vessels and bronchus were erased from the VOI. The regional label was marked in LW settings with a commercially available segmentation software (Yizhun CIPS, version 4.0; http://www.yizhun-ai.com/Content/477572.html) and its pulmonary nodule advanced analysis tool. During the segmentation procedure, the radiographic characteristics were evaluated simultaneously based on NCECT images. To ensure the accuracy, the consensus of segmentation and evaluation was reached by discussion with another experienced thoracic radiologist in case of dilemmatic situations. A third radiologist with 15 years of experience in chest CT interpretation segmented a random set of 20 nodules on NCECT and CECT images independently to assess the interobserver reproducibility of radiomics feature extraction. The features with intraclass correlation coefficients (ICC) > 0.80 were considered in good consistency and then input to the least absolute shrinkage and selection operator classifier (LASSO) classifier to establish the radiomics signature for the distinction between non-invasive and IAC groups.

### Radiomics feature extraction

The radiomics features were extracted from both NCECT and CECT images using pyRadiomics (https://doi.org/10.1158/0008-5472.CAN-17-0339). Each radiomics feature was applied with a Z-score normalization so that the maximum value will be 1.0. Before the feature extraction, the images were resampled to 0.5 mm resolution in all three directions with the cubic convolution interpolation algorithm to normalize the voxel distribution. We used five filters in the experiment to remove the undesired signals.

### Feature selection

The LASSO classifier was used to select the most non-redundant and predictable radiomics features from NCECT and CECT images. Features with nonzero coefficients were rectified by fivefold cross-validation for 100 times.Then we calculated an independent variable correlation matrix of coefficient based on the mean importance of features in previous experiments. The highly correlated features were removed. Patients were randomly segregated into the validation set by step sampling with a ratio of 5:1. Finally, two radiomics signatures built on NCECT and CECT respectively were established for each patient via a linear combination of selected features in the training set. The predictive performance of the two radiomics signature models was evaluated by the Receiver Operating Characteristic (ROC) curve analysis. Area Under the Curve (AUC) value was used to quantify the performance of each model. The optimal signature was selected for further analysis.

### Construction of the radiomics nomogram

Multivariate logistic regression was applied to establish a combined model with independent factors between radiomics signature and radiographic model. The variance inflation factor (VIF) was performed to checkout the collinearity. An individualized prediction nomogram was then constructed based on the multivariate logistic regression model.

### Statistical analysis

The statistical analyses were conducted with R software (version 3.6.2; ACCEPTED MANUSCRIPT 12 http://www.Rproject.org) and SPSS 21.0 (IBM, Chicago, IL, USA). Lasso binary logistic regression was done using the “glmnet” package. Nomogram was performed with the “rms” package. The C-index calculation was done with the “hmisc” package. DCA was performed with the “rmda” package. Multivariate binary logistic regression was done with forward parameter strategy. Categorical variables of radiographic features were compared with Pearson X^2^ tests or Fisher's exact test. Independent t-test was used to assess the difference in radiomics signatures among the training set and the validation set. ROC analysis was performed to evaluate the efficiency of radiomics signatures. The DeLong’s test was applied to test the statistical significance of AUC values. Variance inflation factor (VIF) was used to evaluate the collinearity. The Hosmer–Lemeshow test was performed to assess the goodness-of-fit of the nomogram. A two-sided P value < 0.05 was considered statistically significant.

## Results

### Patients characteristics

The radiographic characteristics of all patients were detailed in Table 1. Of these patients with subcentimeter GGNs, 113 (35.5%) were pathologically diagnosed as AIS, 141 (44.3%) were MIA, while 64 (20.1%) were IAC. In univariate analysis, statistically significant differences were observed in type, shape, vascular change and pleural attachment (P < 0.05). According to the multivariate logistic regression analysis, type, shape and vascular change were statistically significant independent differentiators (P < 0.05). Subsequently, all three parameters were chosen to establish a radiographic model. The AUC of the radiographic model was 0.736 (95% CI 0.669–0.802).

**Table 1:** Radiographic parameters of all patients in non-invasive and invasive groups.

Parameters	Non-invasive	Invasive	Data	P
**Gender**			0.010	0.921
Male	85 (33.5)	21 (32.8)		
Female	169 (66.5)	43 (67.2)		
Age	53.79 ± 11.95	55.98 ± 12.25		0.577
**Location**			7.430	0.115
Right upper lobe	87 (34.3)	11 (17.2)		
Right middle lobe	27 (10.6)	9 (14.1)		
Right lower lobe	38 (15.0)	13 (20.3)		
Left upper lobe	71 (28.0)	20 (31.3)		
Left lower lobe	31 (12.2)	11 (17.2)		
**Type**			18.701	**0.0001**
pGGN	190 (74.8)	30 (48.4)		
mGGN	64 (25.2)	34 (51.6)		
**Shape**			3.553	**0.0001**
Round	168 (66.1)	21 (32.8)		
Irregular	86 (33.9)	43 (67.2)		
**Spiculation**			0.755	0.385
Present	33 (13.0)	11 (17.2)		
Absent	221 (87.0)	53 (82.8)		
**Lobulation**			0.023	0.879
Present	30 (11.8)	8 (12.5)		
Absent	224 (88.2)	56 (87.5)		
**Bubble lucency**			0.190	0.663*
Present	11 (4.3)	2 (3.1)		
Absent	243 (95.7)	62 (96.9)		
**Vascular change**			14.102	**0.0001**
Present	53 (20.9)	28 (43.8)		
Absent	201 (79.1)	36 (56.3)		
**Bronchiole change**			0.690	0.406
Present	23 (9.1)	8 (12.5)		
Absent	231 (90.9)	56 (87.5)		
**Pleural attachment**			6.681	**0.010**
Present	20 (7.9)	12 (18.8)		
Absent	234 (92.1)	52 (81.3)		

Values are presented as no. (%) or mean ± SD. P value is derived from the univariable association analyses between each of the basal variables and the invasive extent.

*Fisher’s exact test.

### Radiomics features selection, signature building

**Figure 1:**
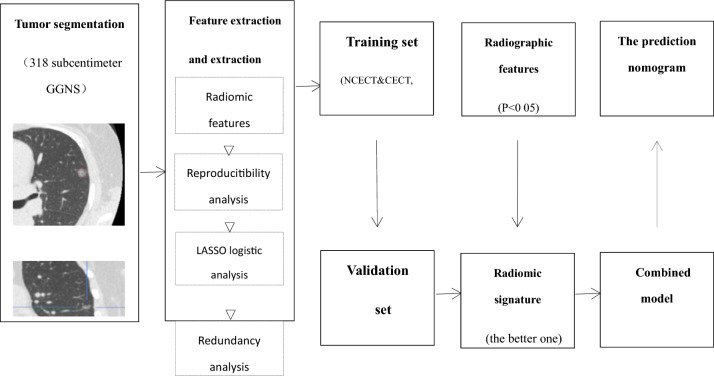
The workflow of radiomics analysis.

254 cases including 203 AIS/MIA and 51 IAC were grouped in the training set. 64 cases (51 AIS/MIA and 13 IAC) were grouped in the validation set. After assessing the reproducibility based on the resegmentation data, a total of 622 features for NCECT and 573 features for CECT with ICC > 0.8 were selected. The workflow of radiomics analyses was indicated in Fig. 1. Finally, 33 potential predictors with nonzero coefficients were conducted into a radiomics signature on NCECT in the training set. While 31 potential predictors were selected to establish a radiomics signature on CECT. The details of the radiomics signature formula were presented in the Supplementary data.

**Figure 2:**
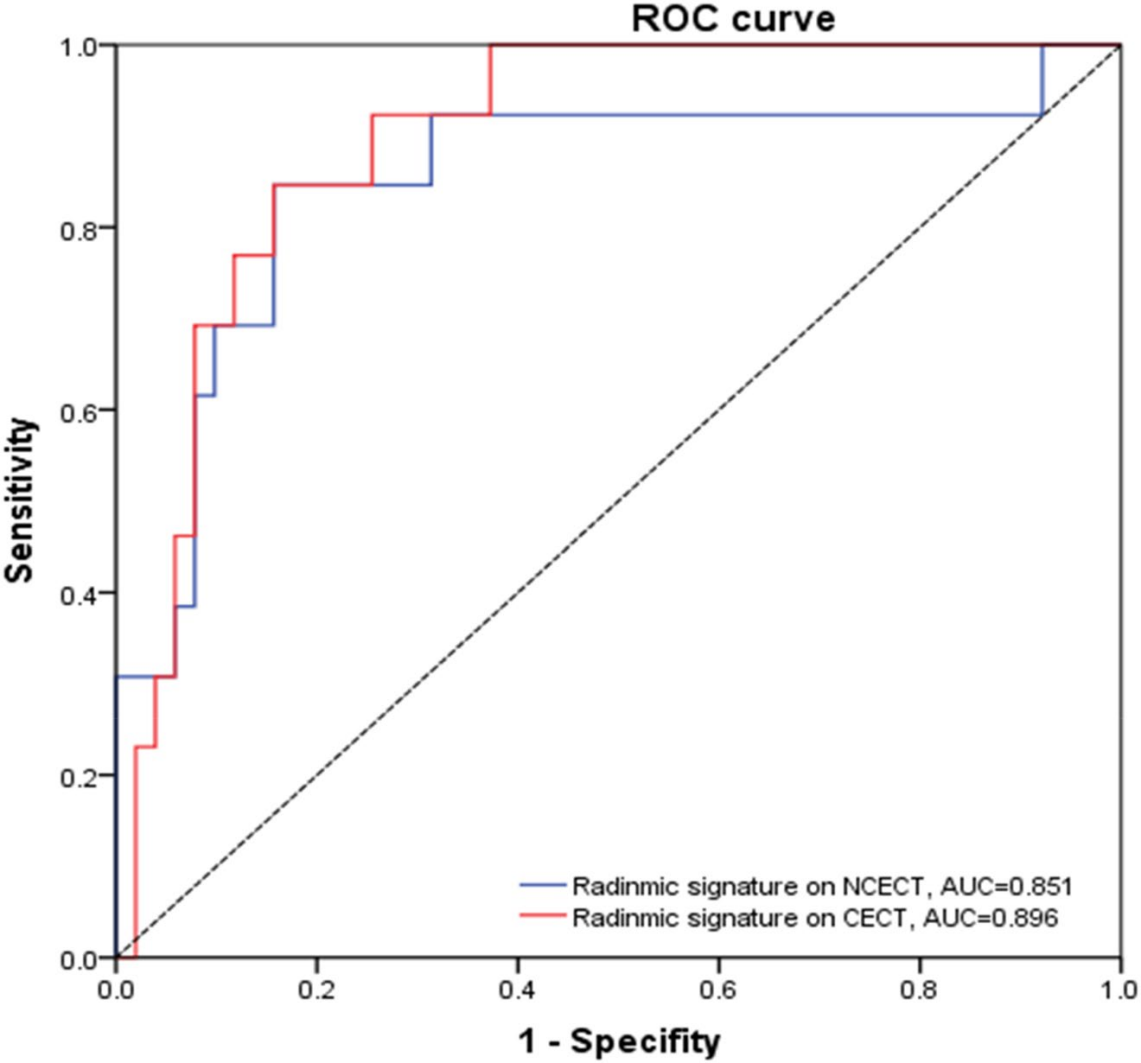
Comparison of ROC curves between radiomics model on CECT (rad line) and radiomics model on NCECT (blue line) in the validation cohort.

The invasive group had significantly higher radiomics signature than the non-invasive group both in NCECT and CECT in the training set (− 1.519 ± 1.334 vs. 0.314 ± 1.075 and − 1.213 ± 0.758 vs. 0.490 ± 1.037, P < 0.0001, respectively). This difference was then verified in the validation set (− 1.755 ± 1.072 vs. − 0.065 ± 1.324 and − 1.164 ± 0.851 vs. 0.430 ± 0.950, P < 0.0001, respectively). The radiomics signature yielded an AUC of 0.868 (95% CI 0.820 to 0.916) for NCECT and 0.917 (95% CI 0.880 to 0.954) for CECT in the training set. Further, we performed DeLong’s test to analyze the difference between the AUC of two radiomics models in the validation set. The result showed that there was a significant difference between two radiomics models (0.851 vs 0.896, P < 0.001) (Fig. 2), which indicated that the radiomics model on CECT performed better than the radiomics model on NCECT. Subsequently, the radiomics model on CECT was selected for further analyses.

### Individualized prediction nomogram and diagnostic validation

**Figure 3:**
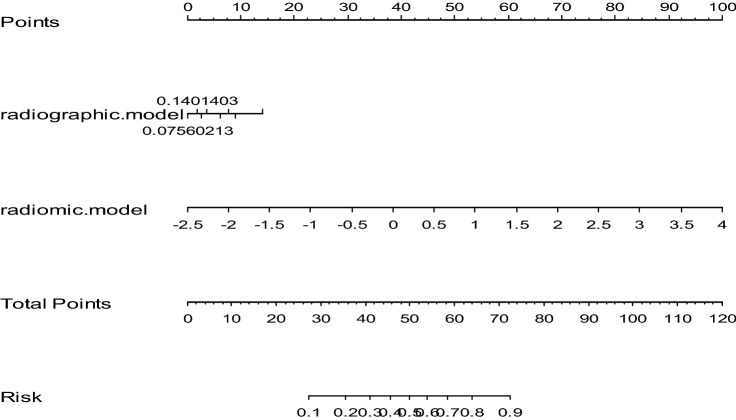
The nomogram was developed incorporating radiomics model on CECT with radiographic signature.

**Figure 4:**
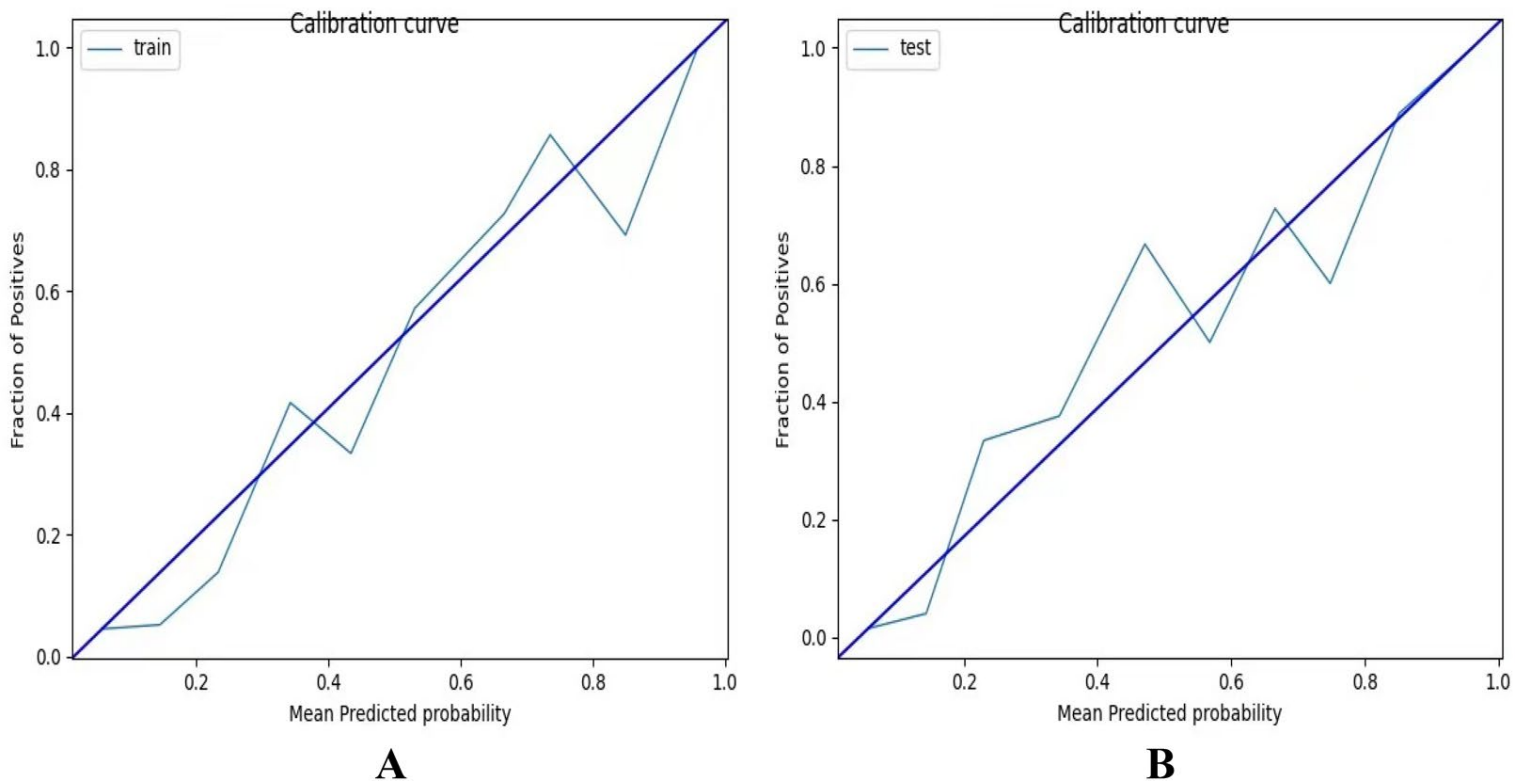
(**A**) Calibration curve of the nomogram in the training set. (**B**) Calibration curve of the nomogram in the validation set.

The VIF between radiographic model and radiomics model was 1.235, which indicated that there was no multicollinearity between the potential factors identified by radiographic analysis and radiomics signature. According to the multivariate logistic regression analysis with forward stepwise selection, both radiographic model and radiomics model were significantly independent differentiators for invasive groups. Subsequently, the two models were integrated to develop an individualized prediction nomogram (Fig. 3). The Hosmer–Lemeshow test yielded no significant statistical difference in the training set and the validation set (P = 0.057 and P = 0.585), which suggested that there was good agreement on the prediction and observation. The calibration curve of the nomogram in the two sets demonstrated in Fig. 4A,B. The C-index of the prediction nomogram was 0.915 in the training set and 0.881 in the validation set.

### Clinical utility

**Figure 5:**
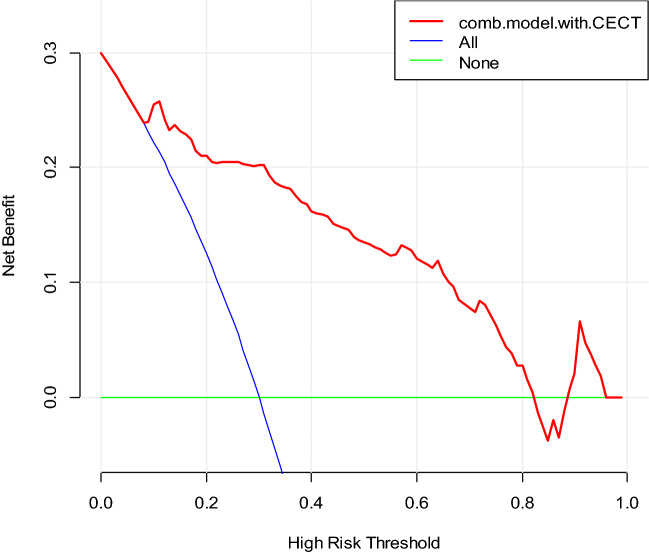
DCA for the individualized prediction nomogram. The y-axis represented the net benefit. The red line represented the nomogram. The blue line represented the hypothesis that all patients with subcentimeter GGNs were invasive lesions. The green line represented the hypothesis that all patients with subcentimeter GGNs were preinvasive lesions. The x-axis represented the threshold probability.

DCA for the individualized prediction nomogram was presented in Fig. 5, which showed that using the nomogram model to predict invasiveness of subcentimeter GGNs added more benefit than the treat-all scheme or the treat-none scenario when the threshold probability of a patient or doctor was 15% to 80%.

## Discussion

Our study developed and validated a radiomics signature built on CECT to discriminate invasive adenocarcinoma from non-invasive ones manifesting as subcentimeter GGNs. Then, we developed a prediction nomogram integrating the radiomics signature with the radiographic model for clinical application. The C-index of the prediction nomogram was 0.935 in the training set and 0.917 in the validation set.

According to the results of radiographic evaluation in our study, mixed GGNs and vascular changes showed more frequently in subcentimeter IAC. In addition, noninvasive lesions demonstrated more round shape. Our observation indicated that for the tumor less than 1 cm, the presence of the solid component was also a prognostic factor for IAC owing to its invasive nature. Moreover, our results showed that the vascular distortion or dilation was associated with the invasiveness of subcentimeter GGN. For that vascular changes within the GGN were more sensitive and accurate in reflecting the histopathologic evolution process^12^. With the morphological basis regarding the difference of the solid component and vascular changes, the prognostic examination of CECT could provide additional information on the subcentimeter GGNs. But previous studies did not focus on the important correlation between CECT and radiomics characteristics in subcentimeter lesions.

Radiomics is an emerging field that offers information on tumour phenotype as well as its microenvironment^13^. A recent study from Coroller et al.^14^ investigated radiomics signature predicting distant metastasis in lung adenocarcinoma. This frontier research was further evaluated by Song’s study on progression-free survival prediction in non-small cell lung cancer patients with EGFR mutations^15^. In our study, we investigated radiomics features based on CECT and NCECT data separately and compared their diagnostic efficiency. Depending on the results, CECT showed better diagnostic capability than NCECT. We eventually selected the CECT data for subsequent analysis. We believed that our result could provide a alternative way of thought in dealing with subcentimeter GGNs. CECT can reveal the increased intratumoral microvascular density along with the deepening of tumor invasion degree and provide extra information on the physiology. These information can be reflected by radiomics analysis. Previous studies provided evidence that radiomics was related to a series of tumor histopathologic process and regarded radiomics as a potential tool for assessing tumor microenvironment noninvasively^16, 17^. In contrast, different opinion showed that texture features extracted from CECT provided no significant benefit in the diagnosis of invasive lung adenocarcinoma compared with NCECT^18^. However, with a larger population and a relatively smaller threshold inclusion criteria in our study, we could provide more references for future relative studies.

To build an efficient radiomics signature, our study calculated higher-order textural parameters from grey-level run-length matrix (GLRLM), grey-level co-occurrence matrices (GLCM), grey-level size zone matrix (GLSZM), neighbouring grey-tone difference matrix (NGTDM) and grey-level dependence matrix (GLDM). These combined application of different matrix features could give a more accurate description of the characteristics of the tumor image in different dimensions, so that reflected the heterogeneity within the tumor^19^. Otherwise, the radiomics features were extracted not only on original CT images but also the images derived from image filters. In this study, we used five different filters: exponential, square, square root, logarithm and wavelet (wavelet-LLL,wavelet-HHH,wavelet-HLL,wavelet-HHL,wavelet-LLH,wavelet-HLH,wavelet-LHL,wavelet-LHH). This filtration process was used to reduce the effect of photon noise while enhancing biologic heterogeneity, which is regarded as a feature of malignancy.

Nomogram emerges as a pragmatic and reliable graphical statistics tool that makes complicated calculations easy^20^. In this study, we built a multi-information nomogram integrating radiomics signature with radiographic model. The combined model demonstrated good calibration and the discrimination power was much better than the traditional image characteristics-based model, relatively higher than previous similar study^21^. However, no significant difference was found in compare with CECT-based radiomics signature (Table 2). We suspected that the radiomics feature Shape, which were incorporated in our analysis, were contribute equally to the radiographic features. With its high-throughput characteristics, it may provide more accurate information in assessment of invasiveness than imaging phenotypes. This may also demonstrate that radiomics features were superior to phenotypic characteristics. To balance the vulnerabilities of radiomics, in this cohort, we incorporated the radiographic features into the nomogram. In order to avoid the multi-collinearity with the first-order characteristics of radiomics, we discarded the continuous variables of radiographic features such as diameter and mean CT value. Our study adopted decision curve analysis to assess the clinical application of our radiomics nomogram-assisted medical treatment of small adenocarcinoma. Decision curve analysis is an appropriate alternative way for multicenter validation to offer insight into clinical consequences on the basis of threshold probability^22^. According to the result in this study, if the risk threshold probability of a patient or doctor was 15% to 80%, our nomogram could add more benefit than a treat-all-patients scenario or a treat-none scheme.

**Table 2:** The performance of the radiographic, radiomics and combined model in all patients.

	Signature score (mean ± SD)		AUC (95% CI)	ACC	SEN	SPE
	Noninvasive group	IAC				
Radiographic model	0.175 ± 0.126	0.304 ± 0.170	0.736 (0.669–0.802)	0.616	0.844	0.621
Radiomics model on NCECT	− 1.566 ± 1.287	0.237 ± 1.129	0.864 (0.816–0.912)	0.730	0.859	0.831
Radiomics model on CECT	− 1.213 ± 0.758	0.490 ± 1.037	0.911 (0.878–0.945)	0.714	0.797	0.906
Combined model	0.114 ± 0.171	0.547 ± 0.305	0.908 (0.871–0.944)	0.701	0.766	0.913

*AUC* area under curve; *ACC* accuracy; *SEN* sensitivity; *SPE* specificity; *PPV* positive predictive value; *NPV* negative predictive value.

Our study has several limitations. First, this retrospective study was performed in a single-center. Potential data selection bias was inevitable. Second, manual segmentation of VOI has a risk of observer bias compared with semi-automatic segmentation. However, previous studies have reported a high degree of intra- and inter-observer reproducibility for manual segmentation and regarded it as the current reference^23, 24^. Third, two different CT vendors were adopted in the current study. Potential interference caused by the different phantom parameters was existed. Future trials with multicenter and standardized CT phantom parameters are needed to further verify the reported findings.

In conclusion, radiomics analysis on CECT images may have the potential to act as imaging correlates for tumor angiogenesis and provide incrementally predictive information for patients with subcentimeter adenocarcinomas. The individualized prediction nomogram derived from radiomics features on CECT in combination with radiographic features could serve as a convenient clinical tool to facilitate early prediction of small adenocarcinomas.

## Supplementary Information

Supplementary Information.
