# Supplementary material for: Radiomics signature on CECT as a predictive factor for invasiveness of lung adenocarcinoma manifesting as subcentimeter ground glass nodules
Source: Sci Rep. 2021 Feb 11;11:3633. doi: 10.1038/s41598-021-83167-3 (PMC7878798; doi:10.1038/s41598-021-83167-3)
Supplement: Supplementary file 1 — Supplementary Information. [file 41598_2021_83167_MOESM1_ESM.docx]

CECT:

**RadScore =** +0.853*cte_square_ngtdm_Busyness+0.834*cte_original_shape_MinorAxisLength+0.737*cte_square_gldm_DependenceVariance+0.704*cte_wavelet-LLL_glcm_JointEntropy-0.594*cte_original_shape_Sphericity+0.591*cte_wavelet-HLL_glszm_SizeZoneNonUniformity+0.556*cte_logarithm_gldm_DependenceNonUniformity+0.545*cte_log-sigma-3-0-mm-3D_firstorder_Energy+0.485*cte_logarithm_glszm_GrayLevelNonUniformity+0.471*cte_exponential_glrlm_RunLengthNonUniformity+0.439*cte_exponential_firstorder_Energy-0.363*cte_log-sigma-3-0-mm-3D_glszm_ZoneEntropy+0.331*cte_exponential_firstorder_InterquartileRange+0.275*cte_original_glrlm_LongRunHighGrayLevelEmphasis-0.274*cte_log-sigma-3-0-mm-3D_gldm_DependenceEntropy-0.274*cte_wavelet-HHH_gldm_GrayLevelNonUniformity+0.252*cte_original_shape_Maximum3DDiameter+0.199*cte_wavelet-LLL_glcm_SumAverage+0.173*cte_wavelet-LLL_glszm_ZoneEntropy+0.158*cte_exponential_firstorder_90Percentile+0.156*cte_log-sigma-3-0-mm-3D_glrlm_RunLengthNonUniformity+0.134*cte_wavelet-LHL_glszm_GrayLevelNonUniformity-0.129*cte_squareroot_glrlm_RunEntropy+0.118*cte_wavelet-HLL_glszm_GrayLevelNonUniformity+0.114*cte_squareroot_glcm_JointAverage-0.109*cte_wavelet-HLH_glrlm_RunLengthNonUniformity-0.106*cte_square_gldm_GrayLevelNonUniformity-0.106*cte_original_glrlm_RunEntropy+0.094*cte_wavelet-HLL_glszm_ZoneEntropy+0.077*cte_squareroot_gldm_DependenceEntropy-1.907

NCECT:

**RadScore =** +1.645*exponential_glrlm_GrayLevelVariance+1.645*exponential_glszm_LowGrayLevelZoneEmphasis+1.260*original_shape_MinorAxisLength+1.142*wavelet-LLL_glrlm_RunEntropy+1.018*exponential_firstorder_90Percentile+0.933*original_shape_Maximum2DDiameterRow+0.731*log-sigma-3-0-mm-3D_glszm_LargeAreaLowGrayLevelEmphasis+0.715*wavelet-LHH_firstorder_Skewness-0.635*wavelet-LLH_firstorder_Median+0.594*square_glcm_Idn+0.468*log-sigma-3-0-mm-3D_glrlm_RunLengthNonUniformity-0.442*wavelet-LHH_firstorder_Median+0.441*wavelet-LHL_glszm_ZoneEntropy+0.440*wavelet-HLH_firstorder_Maximum+0.399*original_glszm_ZoneEntropy+0.373*wavelet-HHL_glrlm_RunEntropy+0.373*logarithm_gldm_LargeDependenceHighGrayLevelEmphasis+0.371*squareroot_glszm_ZoneEntropy+0.357*wavelet-HHH_glrlm_RunVariance-0.316*wavelet-HLH_glcm_Imc1+0.286*square_glrlm_LongRunEmphasis+0.257*wavelet-HLH_ngtdm_Busyness+0.228*wavelet-HHH_glszm_SmallAreaLowGrayLevelEmphasis-0.211*square_glcm_Imc2-0.162*logarithm_firstorder_Mean+0.086*wavelet-LHH_glszm_GrayLevelNonUniformityNormalized+0.027*wavelet-LLH_glcm_MCC-0.024*wavelet-HHL_firstorder_Kurtosis-0.013*square_glszm_SmallAreaHighGrayLevelEmphasis+0.009*wavelet-LLH_gldm_DependenceNonUniformityNormalized-7.037
